# Meningococcaemia causing necrotizing cellulitis associated with acquired complement deficiency after gastric bypass surgery: a case report

**DOI:** 10.1186/s12879-020-05079-3

**Published:** 2020-05-20

**Authors:** Zoe Pletschette, Elodie De Groote, Wesley Mattheus, Charlotte Waxweiler, Jacques Creteur, David Grimaldi

**Affiliations:** 1 Department of Intensive Care, Erasme Hospital, Université Libre de Bruxelles, 808 Route de Lennik, Brussels, Belgium; 2 Department of Infectious Diseases, Erasme Hospital, Université Libre de Bruxelles, Brussels, Belgium; 3 Meningococcal Reference Centre, Sciensano, Brussels, Belgium; 4 Department of Plastic Surgery, Erasme Hospital, Université Libre de Bruxelles, Brussels, Belgium

**Keywords:** Meningococcus; necrotizing fasciitis, Serogroup W, Case report

## Abstract

**Background:**

*Neisseria meningitidis* has rarely been described as an agent of necrotic soft tissue infection.

**Case presentation:**

We report a case of a septic shock with necrotizing cellulitis due to *Neisseria meningitidis* serogroup W, treated by urgent extensive surgical debridement followed by skin grafts. The invasive meningococcal disease occurred together with a complement deficiency, possibly acquired after bypass surgery that took place 1 year before.

**Conclusions:**

Necrotic tissue infections should be considered part of the invasive meningococcal diseases spectrum and should prompt clinicians to look for complement deficiencies. Gastric bypass surgery associated malnutrition may be implicated but further verification is needed.

## Background

*Neisseria meningitidis* are virulent bacteria known for causing fulminant purpura and purulent meningitis, but unusual presentations have been observed. We report here a rare case of necrotizing soft tissue infection (NSTI) related to meningococcaemia associated with a recently acquired complement deficiency.

## Case presentation

In April 2019, a 50-year-old woman was admitted to our Intensive Care Unit for septic shock related to a necrotizing soft tissue infection.

Her medical history mentions a complicated bypass surgery 1 year before followed by severe malnutrition still in need of enteral feeding supplement. Earlier diagnoses include arterial hypertension and discoid lupus erythematosus. She never required immunosuppressive therapy.

**Fig. 1:**
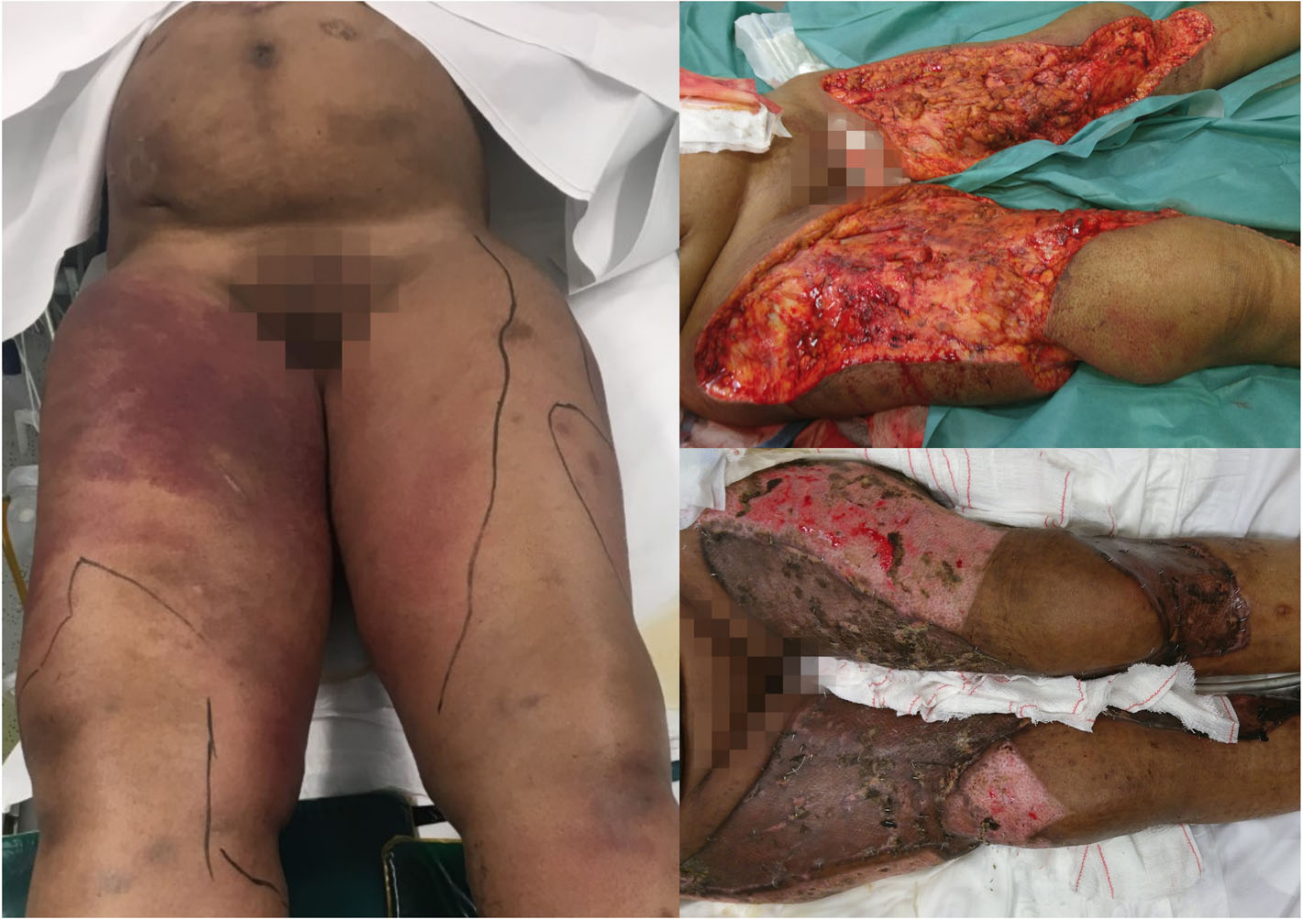
Lower extremities lesions at admission (**a**), after debridement (**b**) and after skin grafting (**c**)

A few hours before admission, the patient developed a sudden intense leg pain, associated with malaise. At the emergency room, she presented hyperaemia and swelling of both anterior thighs and right abdominal flank (Fig. 1a). Blood pressure was 50/35 mmHg, heart rate 140 bpm and temperature 35.3 °C. Arterial blood lactate was 12 mmol/L (*N* < 2 mmol/L).

Intravenous fluids resuscitation was started with high doses of vasopressor (norepinephrine up to 6 mcg/kg/min) and empirical antimicrobial treatment combining piperacillin-tazobactam, amikacin and clindamycin. Septic shock was associated with severe disseminated intravascular coagulation (prothrombin time 30 s, activated platelet time superior to 150 s, platelets 6000/mm3, D -dimers 25,000 mcg/L, fibrinogen 85 mg/dL) and acute kidney injury (creatinine 2 mg/dL, urea 96 mg/dL and oliguria 20 mL/hour).

Urgent surgical exploration of the skin lesions revealed extensive subcutaneous necrosis not encompassing the fascia. The lesions underwent extensive debridement (Fig. 1b).

All surgical sample and blood cultures returned positive for *Neisseria meningitidis*. The first blood culture drawn at admission being so after 9 h. The strain isolated was identified as a serogroup W subtype W: P1.5,2: F1–1: ST-11 (cc11). Genetic comparison based on Core Genome Multilocus Sequence Typing (cgMLST) using the international Neisseria public database for molecular typing (pubMLST) indicated that the isolate belonged to the UK 2013 lineage. The sequence has been deposited into the European Nucleotide Archive (ENA) database and is available under study accession number PRJEB37139.

Antibiotic treatment was de-escalated to benzylpenicillin related to a minimal inhibitory concentration (MIC) measured at 0,06 mg/L.

A lumbar puncture performed at day 2 after coagulation correction returned normal values.

After 52 days spent in ICU with several complications occurring, the patient benefited from successful repair skin grafts (Fig. 1c) and was discharged to the hospital ward 10 days later.

Three months before this necrotizing cellulitis, the patient had undergone an immune status workup by a nephrologist for low-level proteinuria. ANA/ANCAs were negative, while C3 and total haemolytic activity (CH50) levels were low at 55 and 19% respectively. A normal C4 level was found. Complement had been reported normal at the occasion of the earlier diagnostic investigation for discoid lupus erythematosus.

## Discussion and conclusion

Numerous case reports identified *Neisseria meningitidis* as cause of severe cellulitis, more frequently involving the head and neck region [1–5].

Necrotic cellulitis differentiates from Purpura fulminans, which also leads to skin necrosis, but on the basis of confluent petechiae and as a result of endotoxin-related microthrombi [6].

Previously published case reports of *N. meningitidis* related necrotizing soft tissue infections have been treated with extensive surgical debridement just as has been this patient [7, 8]. Although necrosis did not extend beyond the fascia, early surgery in addition to prompt antibiotic treatment may have contributed to the patient’s survival.

The meningococcal strain isolated, which belongs to the serogroup W of the genotype ST-11, is increasingly reported in many European countries with patients presenting abdominal symptoms in contrast to the more conventional presentation of meningococcal infections [9, 10]. This particular ST-11 strain was described as epidemic in South America and then observed in 2009 in the UK and is therefore known as the South American/UK lineage. The original UK strain later evolved through further genetic rearrangements to become the UK 2013 strain [11]. This rising incidence led to the promotion of the ACWY vaccination rather than the MenC vaccination (recommended since July 2019 in Belgium).

Invasive meningococcal diseases (IMD) are favoured by complement deficiencies. Inherited deficiencies involving alternate pathway (C3 [12], factor D, properdin [13]), or terminal pathway components (C5 through C9) [14–16] are well-described risk factors for IMD in childhood [17].

Acquired deficiency in C5, induced by the therapeutic monoclonal antibody eculizumab (an inhibitor of C5 cleavage), has also been shown to favour IMD [18].

Hypocomplementemia can be due to immune complex formation in antibody-mediated immune diseases such as cryoglobulinemia, systemic lupus erythematosus and endocarditis. However, to our knowledge, this phenomenon has not been linked to an increased susceptibility to IMD [17].

In our case, the CH50 and the C3 were abnormally low 3 months before the IMD though normal several years earlier. We hypothesize that the complement deficiency was acquired following the complicated gastric bypass surgery. This is suggested by the study of Gómez-Abril et al. [19] whose systematic exploration of immunological and laboratory abnormalities following bypass surgery demonstrated low levels of C3.

While vaccination against *N. meningitidis* in complement deficient persons is widely recommended [20], it is not clear at this stage how frequent hypocomplementemia is to be found after gastric bypass surgery since our case is likely the first one described. Vaccination of such patients could be offered broadly once complement deficiencies is demonstrated regularly.

In conclusion, we report a rare case of *N. meningitidis* related necrotizing cellulitis, an entity different from fulminant purpura. Meningococcemia was possibly favoured by an acquired classical pathway complement deficiency following a complicated gastric bypass entailing severe malnutrition.

*Neisseria meningitidis* should be considered among the causes of necrotizing cellulitis. Whether gastric bypass surgery associated malnutrition impairs complement function deserves further confirmation.

## Data Availability

All data generated or analyzed during this study are included in this published article.

## Abbreviations

NSTI: Necrotizing soft tissue infection
cgMLST: Core genome multilocus sequence typing
pubMLST: Public multilocus sequence typing
ENA: European Nucleotide Archive
MenC: Meningococcal C vaccine
MenACWY: Meningococcal ACWY vaccine
UK: United Kingdom
MIC: Minimal inhibitory concentration
ICU: Intensive Care Unit
ANA: Anti-nuclear antibody
ANCA: Anti-neutrophil cytoplasmic antibody
IMD: Invasive meningococcal diseases
MBL: Mannan-binding lectin
